# Distinctive Features of the XBB.1.5 and XBB.1.16 Spike Protein Receptor-Binding Domains and Their Roles in Conformational Changes and Angiotensin-Converting Enzyme 2 Binding

**DOI:** 10.3390/ijms241612586

**Published:** 2023-08-09

**Authors:** Tej Sharma, Bernard Gerstman, Prem Chapagain

**Affiliations:** 1 Department of Physics, Florida International University, Miami, FL 33199, USA; 2 Biomolecular Sciences Institute, Florida International University, Miami, FL 33199, USA

**Keywords:** SARS-CoV-2, receptor-binding domain, spike protein, omicron, XBB.1.5, XBB.1.16, molecular dynamics, structural changes, mutations, computational

## Abstract

The emergence and the high transmissibility of the XBB.1.5 and XBB.1.16 subvariants of the SARS-CoV-2 omicron has reignited concerns over the potential impact on vaccine efficacy for these and future variants. We investigated the roles of the XBB.1.5 and XBB.1.16 mutations on the structure of the spike protein’s receptor-binding domain (RBD) and its interactions with the host cell receptor ACE2. To bind to ACE2, the RBD must transition from the closed-form to the open-form configuration. We found that the XBB variants have less stable closed-form structures that may make the transition to the open-form easier. We found that the mutations enhance the RBD–ACE2 interactions in XBB.1.16 compared to XBB.1.5. We observed significant structural changes in the loop and motif regions of the RBD, altering well-known antibody-binding sites and potentially rendering primary RBD-specific antibodies ineffective. Our findings elucidate how subtle structural changes and interactions contribute to the subvariants’ fitness over their predecessors.

## 1. Introduction

The emergence of the COVID-19 pandemic caused by SARS-CoV-2 has resulted in a devastating toll on global health, with over six million fatalities and over half a billion confirmed cases [1]. The SARS-CoV-2 virus consists of a positive-strand RNA, protected by a protein capsid that is enveloped by a lipid bilayer in a spherical shape. The virus’ spike protein that protrudes from the lipid bilayer is critical in host cell invasion and is often the target of antibodies [2,3,4,5,6]. The receptor recognition mechanism of SARS-CoV-2 involves the receptor-binding domain (RBD) of the spike protein that binds to the host cell’s angiotensin-converting enzyme 2 (ACE2), and it is a critical initial step of virus entry to the host cell [7,8]. RBD mutations can enhance its ability to bind to the host cell’s ACE2 and therefore improve the virus’ ability to enter and impact the host cell. In addition, genetic variations in ACE2 sequences in different human populations can also alter the RBD binding [9], affecting the global spread of SARS-CoV-2 variants.

As of January 2023, the omicron variant has given rise to five distinct lineages, including BA.1, BA.2, BA.3, BA.4, and BA.5. Among these, BA.1, BA.2, and BA.5 have gained prominence globally [10]. However, there has been a recent surge in the incidence of omicron BQ.1 and its sublineages BQ.1.1 and XBB, which are rapidly replacing previously dominant strains like BA.5 [11]. These sublineages carry multiple mutations in the spike protein’s RBD, a key target for SARS-CoV-2 vaccine design and immunotherapy. In addition, studies have shown that these sublineages are more resistant to the humoral immunity conferred by vaccination, prior infection, or therapeutic monoclonal antibodies than earlier omicron strains such as BA.2 and BA.5. The XBB.1.5 and XBB.1.16 variants that evolved from XBB have been deemed the most proficient and transmissible strains to date. Following their initial detection in late 2022, they demonstrated greater transmission rates and rapid proliferation, outpacing BQ.1.1. The XBB variants have emerged as the dominant lineages of new cases in the United States in recent updates. As SARS-CoV-2 adjusts to infection- or vaccine-induced immunity and control measures, it is generally expected that future variants may evolve with the features of high infectivity, high immune escape, and low virulence [12] and/or acquire fitness for multi-variants in co-existence [13], referred to as variant soup [14]. However, the emergence of new variants still poses a significant risk to global health. In this work, we have performed a computational analysis of the two new subvariants of omicron—XBB.1.5 and XBB.1.16, which are highly transmissible—and elucidated the molecular mechanisms by investigating the RBD opening as well as the ACE2 binding.

**Figure 1:**
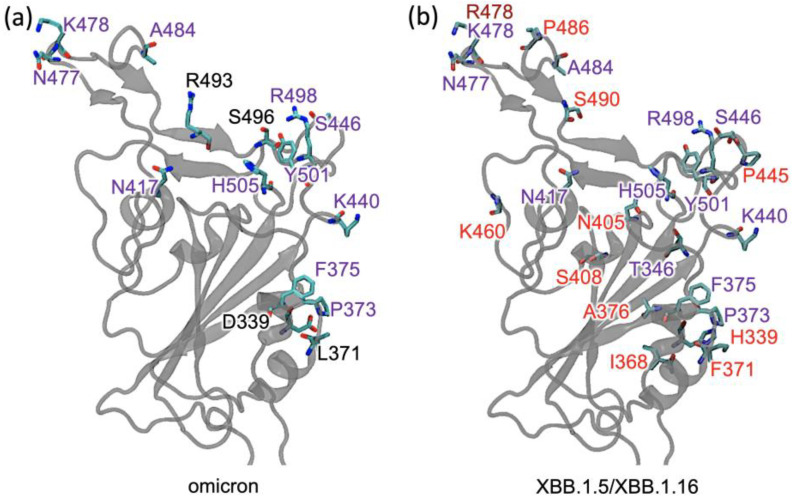
The receptor-binding domain (RBD) of different variants: (**a**) Mutations in omicron are labeled in black or purple. The mutations labeled in purple are also present in XBB.1.5. (**b**) The additional mutations in XBB.1.5 and XBB.1.16 are labeled in red, and the mutation K478R in XBB.1.16 is labeled in brown.

The omicron subvariants, XBB.1.5 and XBB.1.16, have the highest number of mutations in the RBD, with a total number of 22 mutations when compared to the RBD of ancestral SARS-CoV-2. Like previous variants, their mutations enhance their transmissibility [15,16] and their ability to evade antibodies [17,18,19,20]. The XBB lineage arose from the natural co-infection of a human host by two omicron subvariants, BA.2.10.1 and BA.2.75 [21], which gradually replaced other subvariants worldwide and raised global concerns. The XBB.1.5 is a sublineage of the XBB variant, originating from the recombination of two BA.2 sublineages with the S486P mutations (Figure 1) at an evolutionary hotspot [22,23]. The mutation S486P in the spike protein has been found to enhance the infectivity of SARS-CoV-2 by increasing its binding affinity to the ACE2 receptor in human cells [22,23].

Another variant, XBB.1.16, was initially identified in India with a single mutation (K478R) in the RBD of XBB.1.5 (Figure 1). This mutation may be associated with a surge of COVID-19 cases in India and the United States. Earlier studies demonstrated that K417N, Q498R, and N501Y mutations in the RBD region raise the variant’s ability to bind to the human ACE2 receptor. Mutations in residue 484 in the loop area have been associated with the virus’s ability to evade the immune system field [17,18,24,25].

Binding to ACE2 requires the RBD to switch from the closed-form to the open-form configuration. Changes in the RBD interactions in the closed form of the XBB variants may affect the transition to the open form. The spike protein’s flexibility between the down and up conformations is enabled by three hinges within the stalk domain involving residues that are highly conserved across variants. Structural analyses of the spike trimer with or without ACE2, including those with all RBDs in the down conformation or with only one RBD in the up conformation, have yielded valuable insights into the intricate mechanism governing cellular attachment [26].

In the present study, we performed molecular dynamics studies to analyze the structural and functional alterations induced by mutations in the receptor-binding domain (RBD) of the XBB.1.5 and XBB.1.16 variants of SARS-CoV-2. We elucidate the molecular mechanism underlying the binding affinity of these variants to the human cell receptor ACE2 compared to the wild-type (WT), delta, and omicron variants. Our results revealed that the closed, down-form configuration of the RBD has fewer stabilizing bonds in XBB compared to omicron. We also found that, compared to XBB.1.5, the XBB.1.16 variant exhibits a modest increase in RBD–ACE2 affinity, likely due to the K478R mutation facilitating more interactions for other amino acids in the loop region of the RBD. In addition, XBB conformational changes in an RBD motif region may allow antibody evasion. These changes in the RBD structure and RBD–ACE2 interactions may facilitate more efficient transmissions of the XBB.1.16 variant globally.

## 2. Results and Discussions

The attachment of the virus to the host cell occurs by opening the spike protein’s receptor-binding domain (RBD) from a closed (or down) to an open (or up) conformation (as shown in Supplementary Figure S1), followed by the binding of the up conformation RBD to the host’s cell-surface receptor ACE2. We investigated the effects of the XBB mutations on both of these steps. We found that both variants showed weakened interactions, suggesting a potentially easier switch from the down configuration to the up configuration.

### 2.1. RBD Interactions within the Spike Protein in Its Closed Form

**Figure 2:**
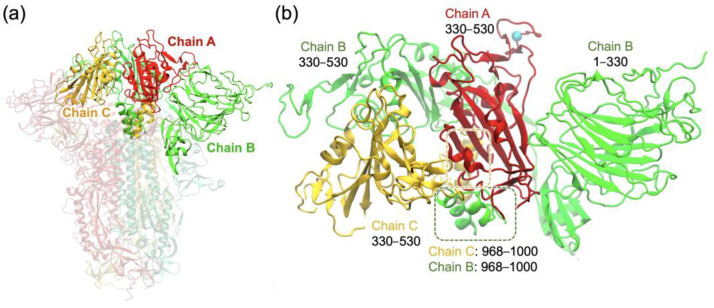
(**a**) XBB.1.5 spike protein trimer, showing the three chains A, B, and C in a closed form with the RBDs in the down positions. (**b**) Truncated trimer system consisting of the RBD of chain A (red) surrounded by other chain segments considered in the simulation system. Residue R478 of the chain A RBD is shown as a cyan sphere.

As shown in Figure 2a, the spike protein consists of three chains (labeled as Chain A, Chain B, and Chain C). To investigate the effects of the XBB.1.5 or XBB.1.16 mutations on the RBD conformational changes from the closed to the open configuration, we performed MD simulations of the RBD together with its surrounding environment in the closed structure of the spike trimer and examined the RBD interactions. We are interested in the stability of the RBD in the closed-form structure of the XBB.1.5 and XBB.1.16 variants compared to omicron.

To determine the stabilizing inter-domain hydrogen bonds associated with the RBD in a closed-form spike trimer, we set up the system similarly to our previous study on the omicron variant [25] and employed a truncated trimer system (Figure 2b) to reduce computational time. The helical segments highlighted within the dashed boxes represent noncontiguous segments of chains B and C that interact with the RBD of chain A. This system consisted only of the domains that directly interacted with the RBD of chain A, which encompassed residues 330–530 of chain A (RBD), 30–530 and 968–1000 of chain B, and 330–530 and 968–1000 of chain C, as shown in Figure 2b. To ensure stability during the simulations, the domains surrounding the RBD of chain A were harmonically constrained using harmonic forces on all C_α_ atoms greater than 12 Å away from the RBD, allowing RBD flexibility within the trimer while maintaining the domain integrity to emulate the full trimer. Our previous work [25] showed that the truncated system produces results of the hydrogen bonding and other closed-form RBD interactions consistent with those for the full spike trimer in the RBD-down conformation [26]. For the purpose of investigating the RBD interactions in the closed-form spike trimer, we used only one system, XBB.1.5, since the only RBD mutation K478R in XBB.1.16 is relatively far from the other chains (shown in Figure 2b) and does not affect the surrounding interactions in the closed-form spike trimer.

**Figure 3:**
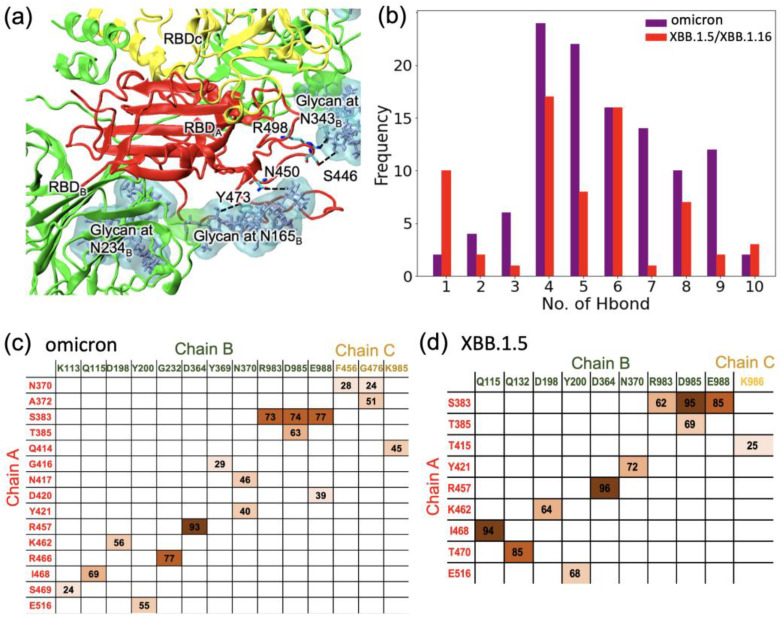
(**a**) The major hydrogen bonds between the RBD of chain A and glycans on chain B in the closed-form spike trimer in XBB. (**b**) The likelihood (frequency) of finding a specific number of hydrogen bonds between the RBD of chain A and glycans on chain B in the closed-form spike trimer. (**c**,**d**) Occupancies of specific Hbonds between residues on chain A and residues on either chain B or chain C: (**c**) omicron and (**d**) XBB. Hydrogen bonding data for omicron in (**c**) were obtained from our earlier work [25] for comparison. Darker shading is used for higher frequency.

We performed a 100 ns simulation for the XBB.1.5 closed-form RBD in the truncated trimer form and analyzed the last 50 ns of the trajectory to determine the hydrogen bonds with surrounding residues and N-glycans. We then compared the results of the XBB.1.5 simulation with those for omicron that we obtained in our previous work [25]. Figure 3a displays the location of the RBD of chain A (red) relative to the surrounding chains in the closed-form spike trimer and highlights important hydrogen bonds with N-glycans on chain B. Figure 3b displays the frequency of a specific number of hydrogen bonds (Hbonds) between chain A and glycans N165, N234, and N343 in the RBD of chain B. From Figure 3b, we see that XBB.1.5 or XBB.1.16 has, on average, fewer Hbonds between the chain A RBD and the chain B glycans; this implies slightly reduced stability of the closed-form RBD in XBB variants compared to omicron and therefore an easier transition to the RBD up structure for XBB.1.5 or XBB.1.16. It is to be noted that the glycan interactions in these variants are still significantly enhanced compared to WT [25], indicating the importance of glycan gating in these newer variants.

In addition to examining the Hbonds between the chain A RBD and the glycans on chain B, we also investigated the hydrogen bonding between the chain A RBD and residues on chain B or chain C. The occupancies are given in the matrices in Figure 3c for the omicron variant and in Figure 3d for XBB.1.5. The sum of the occupancies for XBB.1.5 is lower by 15% compared to the sum of the occupancies for omicron. As in our earlier work [25] on omicron, we found that many Hbonds observed in the omicron RBD were not present in XBB.1.5. Specifically, a strong Hbond between R466 (chain A)–G232 (chain B) in omicron was absent in XBB.1.5. In addition, most of the Hbonds between chain A and chain C in omicron were not present in XBB.1.5. Taken together, compared to omicron, the XBB.1.5 RBD in the closed form was observed to have weaker interactions with the surrounding residues as well as the glycans, implying that the closed-form conformation of XBB.1.5 is less stable than in omicron, and this may allow an easier transition to the open conformation for XBB.1.5.

### 2.2. RBD–ACE2 Interactions in XBB.1.5 and Comparison to the Variants

**Figure 4:**
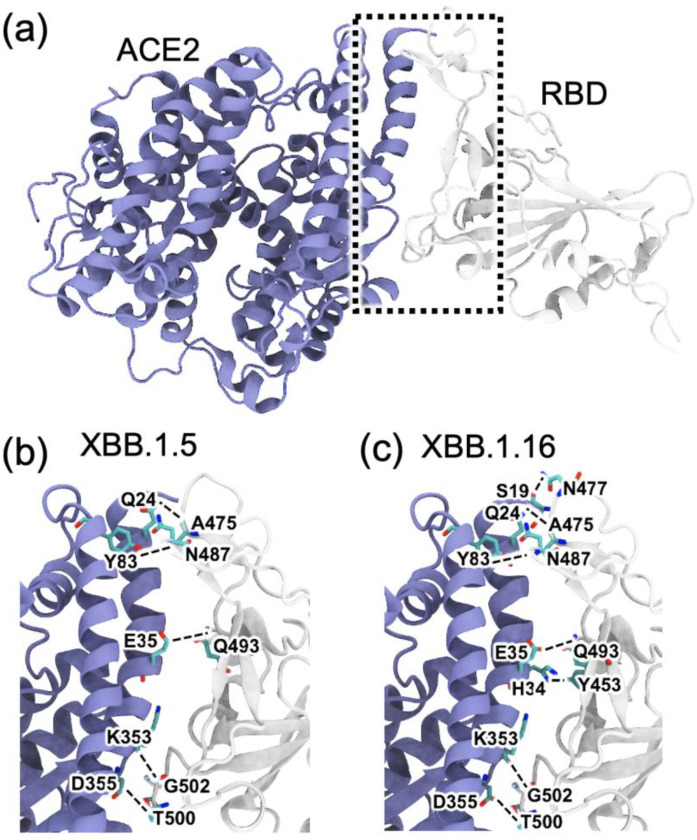
Snapshots of the RBD (gray)–ACE2 (blue) complexes obtained from 300 ns MD simulations for the XBB.1.5 and XBB.1.16 variants. (**a**) Representative RBD–ACE2 complex for XBB.1.5 at 300 ns, with interfacial region shown in a dotted box. (**b**,**c**) Expanded views showing the interfacial hydrogen bonds (dashed lines) formed between the RBD and ACE2 in (**b**) XBB.1.5 and (**c**) and XBB.1.16.

We conducted an analysis of the dynamics of the RBD–ACE2 complexes for XBB.1.5 and XBB.1.16 and observed significant differences in the association of residues in the interfacial region. Compared to the WT, XBB.1.5 had 22 mutations in the RBD, 11 were also present in omicron, and 11 were additional mutations. XBB.1.16 had the same RBD mutations as XBB.1.5 except at residue 478, which lies in the loop region; XBB.1.5 had T478K, while XBB.1.16 had T478R. The loop region involving residue 478 was involved in ACE2 binding. The mutations in the RBD of the XBB sublineages XBB.1.5 and XBB.1.16 are described in Figure 1, and the RBD–ACE2 complex is shown in Figure 4, with the interfacial interacting residues highlighted.

**Figure 5:**
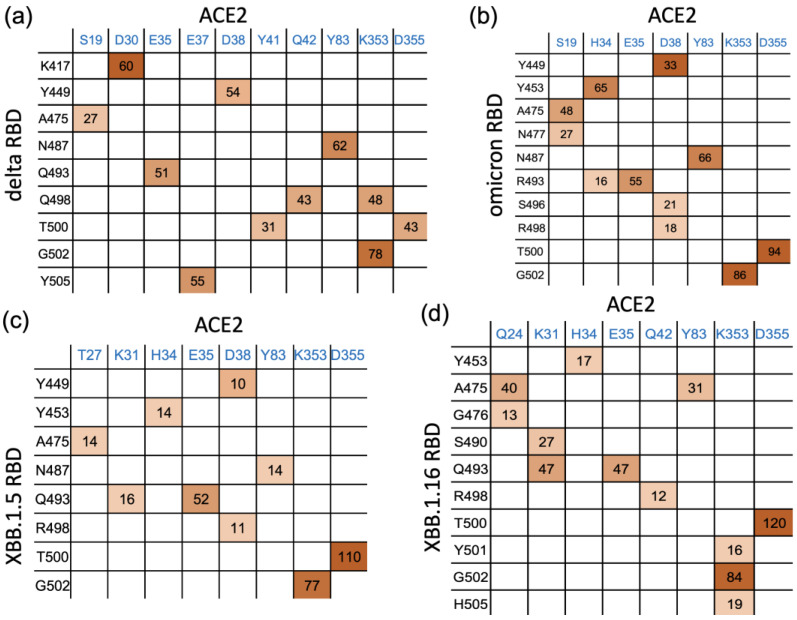
Interaction matrix for the interfacial residue pairs contributing to RBD–ACE2 hydrogen bonding: (**a**) delta and (**b**) omicron. (**c**) XBB.1.5 and (**d**) XBB.1.16 variants. The percentage of time (occupancy) that each hydrogen bond remains intact is given in the interaction matrix. Darker shading is used for higher frequency.

We analyzed the interaction patterns between the RBD and ACE2 during 300 ns of simulations and observed that the mutated residues in XBB.1.5 and XBB.1.16 significantly affect the interfacial interactions. We determined the number of hydrogen bonds formed in the last 100 ns of the 300 ns simulations and identified the major hydrogen-bonding residue pairs. Figure 5 highlights some of the residues involved in hydrogen bonding in the RBD–ACE2 complex for each variant. The percentage of time that interfacial hydrogen bonds existed for during the last 100 ns of the simulations is shown in Figure 5 for the delta, omicron, XBB.1.5, and XBB.1.16 variants. Figure 5 shows that in XBB.1.5 and XBB.1.16, the number and pattern of interactions between the RBD and ACE2 noticeably differ compared to delta and omicron. The sum of the hydrogen bonds’ occupancy percentage is much lower in XBB.1.5 and noticeably improves in XBB.1.16. We note that the hydrogen bond analysis presented here was performed for the 200 ns simulations of the delta and omicron variants starting from their Cryo-EM structures, whereas the RBD–ACE2 complexes for XBB.1.5 and XBB.1.16 were obtained by introducing the mutations to the omicron RBD. Therefore, as the mutated structures relax during the simulations, changes in the hydrogen bonding pattern are observed. Figure S2 shows the number of RBD–ACE2 hydrogen bonds as a function of time for the 300 ns simulation of each mutated complex. The first 150 ns of XBB.1.5 shows a relatively high hydrogen bond occupancy as in omicron, but the structure relaxes to a lower hydrogen bond occupancy after 150 ns. The primary hydrogen bonds G502-K353 and T500-D355 are present in all the variants, with lower occupancies in delta and omicron. While some hydrogen bonds in the delta and omicron complexes disappear in the mutated variants, one major hydrogen bond, T500-D353, strengthens in XBB.1.5 and XBB.1.16. Compared to XBB.1.5, the mutation in XBB.1.16 leads to additional interactions, such as S490-K31, H505-K353, and Y501-K353. To confirm the differences in the interfacial interactions due to a single mutation in XBB.1.5 vs. XBB.1.16, we performed one additional run (run 2) for XBB.1.5 and two additional runs (run 2 and run 3) for XBB.1.16, starting from the 200 ns time frame of the XBB.1.5 simulation. In these additional runs, as shown in the number of hydrogen bonds in Figure S2 as well as in the interaction matrix in Figure S3, the overall hydrogen bonding in XBB.1.16 is consistently higher compared to XBB.1.5.

Other minor differences among the variants were also observed: Y453 makes Hbond pairs with H34 in omicron, XBB.1.5, and XBB.1.16 but not in delta. Hydrogen bond analysis also shows a new Hbond (G476-Q24) in XBB.1.16 that is absent in other variants and a stronger A475-Q24, presumably facilitated by the R478 mutation (Figure 1), in XBB.1.16. Such additional hydrogen bonds at the RBD–ACE2 interface in XBB.1.16 compared to XBB.1.5 suggest the formation of a more stable RBD–ACE2 complex for the XBB.1.16 variant.

Additionally, we noticed that the glycans at position N90 in the interface have a relatively strong interaction, with >100% hydrogen bond occupancy, for XBB.1.5 and XBB.1.16 vs. 80% in omicron. In addition to the hydrogen bonds, van der Waals, as well as hydrophobic interactions near the loop region, may play a role in stabilizing the complexes. These results provide information regarding the intermolecular interactions and structural changes of the RBD–ACE2 complexes, shedding light on the differences in ACE2 binding among these variants.

### 2.3. Structural Changes in the RBD Loop Region and a Motif Region

**Figure 6:**
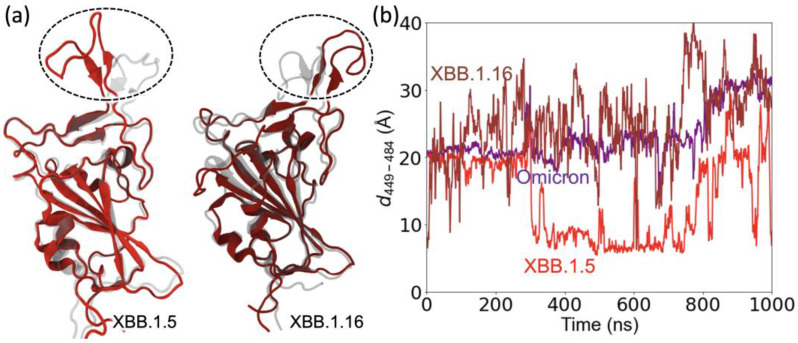
Changes in the position of the loop and motif regions of XBB.1.5 compared to omicron. (**a**) XBB.1.16 compared to XBB.1.5. (**b**) The C_α_–C_α_ distance between the RBD loop residue 484 and RBD residue 449 outside the loop.

SARS-CoV-2 mutations can alter the viral protein structure [27] in a way that helps evade antibodies [28]. To investigate this aspect of mutations, we investigated structural changes in the RBD loop region comprising residues 470 to 491 and a motif region comprising residues 364–376. The loop region contains five mutations compared to WT: S477N, T478K, E484A, S486P, and F490S and a disulfide bond between residues C480 and C488 in XBB.1.5 as well as the same mutations for XBB.1.16, except for T478R. The motif region contains five mutations compared to the WT (L368I, S371F, S373P, S373F, and T376A) that are the same for both XBB.1.5 and XBB.1.16. We performed 1000 ns MD simulations on the RBD of chain A for both XBB.1.5 (Video S1) and XBB.1.16 (Video S2). We used the omicron structure as the initial configuration for the XBB.1.5 simulation. To focus on the differences between XBB.1.5 and XBB.1.16, we used the final configuration from the XBB.1.5 simulation as the initial structure for the 1000 ns XBB.1.16 simulation. The initial and final configurations of the XBB.1.5 (left) and XBB.1.16 (right) are shown in Figure 6a, with the initial configurations shown in gray. Supplementary Figure S4 shows the differences in the bonding pattern that allow changes in the loop structure during the simulations of XBB.1.5 and XBB.1.16.

To examine the reorientation of the RBD loop, we calculated the distance as a function of simulation time between two residues, one from outside the loop (residue 449) and the other from a residue within the loop (residue 486). Figure 6b shows that this distance remains relatively stable for omicron. However, the interaction pattern changes in XBB.1.5 due to the mutations (Figure S4a,b), causing the loop to reorient (Figure 6a). A major change was observed at ~300 ns for the distance between the two residues in XBB.1.5, followed by large fluctuations, as shown in Figure 6b. Likewise, the floppiness of the XBB.1.16 loop results in large fluctuations in the residue separation and a noticeable change in its configuration compared to XBB.1.5. The reorientation of the RBD loop region (residues 470–491) in the XBB variants allows the loop to make more Hbonds with ACE2 compared to omicron.

**Figure 7:**
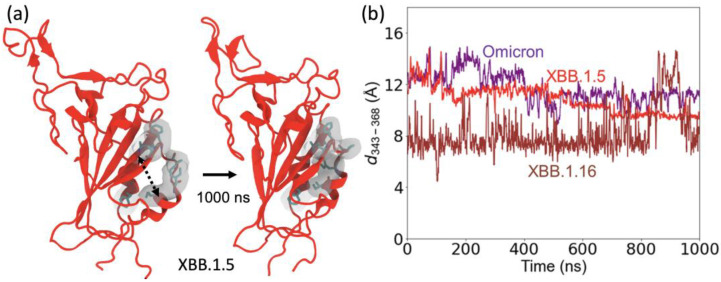
Structural changes in the motif region (residues 364 to 376). (**a**) The structure of XBB variants (shown here for XBB.1.5), starting from the omicron configuration, changes due to the hydrophilic to hydrophobic mutations in the motif region enabling hydrophobic interactions between the two small helices. Hydrophobic residues in the motif are highlighted on a gray surface. (**b**) The C_α_–C_α_ distance between the RBD loop residue 343 and RBD residue 368 to represent the structural changes in the two helices.

We also investigated the change in the structure of the motif regions (residue 364–376) in the XBB.1.5 and XBB.1.16 variants. The motif region of these XBB variants contains five mutations compared to the WT. These include S373P and S375F hydrophilic to hydrophobic mutations, which are also present in omicron, and two additional hydrophilic to hydrophobic mutations S371F and T376A. These hydrophilic to hydrophobic mutations produce a conformational change in which a small outer helix in the motif region moves closer to an inner helix and creates a hydrophobic cluster with A435 and W436 in the inner helix (Figure 7a).

To explore the structural changes in the motif region, we calculated the distance between two residues, 343 on one helix in the motif and residue 368 on a nearby helix in the motif, as shown in Figure 7b. The starting configuration of XBB.1.5 is the same as the omicron structure. During the MD simulation, the separations between the helices decrease in XBB.1.5 and XBB.1.16 compared to those in omicron. The XBB.1.16 curve in Figure 7b shows that the separation between these motif helices decreases significantly for the XBB.1.16 variant compared to XBB.1.5. This decrease is due to additional hydrophobic interactions involving S371F and T376A in the XBB.1.5 and XBB.1.16 variants. Because this motif (especially residues 371 and 375) region is one of the major binding sites for antibodies [25], these structural changes allow evasion from antibody binding to these specific sites.

## 3. Materials and Methods

### 3.1. Molecular Dynamics Simulations

Molecular dynamics (MD) computations were performed with a similar procedure as in our previous work [25,29,30]. Briefly, we employed NAMD2.14 [30,31] with the CHARM36m force field [32]. For each system, the protein was placed in a box of TIP3 water model [33,34], and K^+^ and Cl^−^ ions were added to make a neutral system with the ion concentration of 150 mM. The final setups of the prepared systems are summarized in Table S1. Each simulation started with a 10,000-step minimization and 2 ns equilibration, followed by production runs conducted with a 2 fs time step at 303.15 K temperature and 1 atm constant pressure. Langevin temperature coupling (with 1 ps^−1^ friction coefficient) and the Nose–Hoover Langevin piston method (piston period of 50 fs and decay period of 25 fs) were used for controlling the temperature and pressure, respectively [35,36,37]. The covalent bonds with hydrogen atoms were fixed using the SHAKE algorithm [38]. The nonbonded cut-off was set to 12 Å with a switch distance of 10 Å. The particle mesh Ewald method [39,40] was used for electrostatic calculations. All simulations were performed at a neutral pH (i.e., without changing the charge state). Visual Molecular Dynamics (VMD) 1.94 [41] was used to visualize the structures and simulation trajectories and to calculate the hydrogen bonding, with a 3.5 Å donor–acceptor (heavy atoms) distance cut-off and a 30° threshold angle (heavy atom–hydrogen–heavy atom).

### 3.2. System Preparation

All systems, summarized in Table S1, were prepared with the CHARMM-GUI web server [42,43] using the Solutions Builder tool. The fully glycosylated truncated trimer system for XBB.1.5 and XBB.1.16 was created by selecting an RBD and surrounding environment and adding specific XBB.1.5/XBB.1.16 mutations (shown in Figure 1) to the WT Cryo-EM structure of the spike protein trimer (PDB ID: 6VXX), obtained from Amaro lab’s COVID-19 Data Sets (initial frame of the spike protein trimer simulation). The spike residues N165, N234, and N343 as well as the ACE2 residues N53, N90, N103, N322, and N546 were glycosylated. The XBB.1.5 mutations in the RBD (residues 330–530) are G339H, R346T, L368I, S371F, S373P, S375F, T376A, D405N, R408S, K417N, N440K, V445P, G446S, N460K, S477N, T478K, E484A, S486P, F490S, Q498R, N501Y, and Y505H. The mutations are the same in XBB.1.16 except for T478R.

The RBD–ACE2 complex structure for omicron was obtained from the RCSB with PDB ID: 7T9L [44]. This was used to create both the RBD–ACE2 systems as well as the RBD-only system for omicron, XBB.1.5, and XBB.1.16.

## 4. Conclusions

As SARS-CoV-2 continues to respond to vaccines and control measures, it is accumulating many mutations that provide a better fit for the virus to spread and continue its existence. As such, the XBB.1.5 and XBB.1.16 mutations enhance their transmissibility and their ability to evade antibodies, establishing their dominance over other variants for the time being. In this work, we investigated the effects of the mutations in these variants on the two crucial steps in viral entry to the host cell, (1) the conformational transition of the spike protein from an RBD-down to RBD-up conformation and (2) the binding of the RBD-up structure to the host cell surface receptor ACE2. We found that, compared to omicron, XBB.1.5 (or XBB.1.16) has fewer interactions with its surrounding environment, both with the glycans as well as the amino acid residues, in the closed-form (all RBD-down) configuration of the spike trimer. This suggests that these XBB variants potentially switch to open, up-configuration more easily and present the RBD to bind ACE2.

Our investigations of the RBD–ACE2 complex show that compared to the omicron variant, the XBB.1.5 and XBB.1.16 variants have a different pattern of the RBD–ACE2 interactions, with weakened hydrogen bonding for some interacting pairs but strengthened bonding for others, showing an agile interface. Also, the single mutation K478R in XBB.1.16 compared to XBB.1.5 appears to allow a stronger ACE2 bonding, which can facilitate and enhance the viral RBD attachment to the host cell. We also found that an RBD motif region shows a modest structural change due to the presence of additional hydrophilic to hydrophobic mutations in the XBB.1.5 and XBB.1.16 compared to omicron. Because the residues in the motif region, as well as in the flexible loop region, are major binding sites for antibodies, these mutation-induced structural changes may reduce antibody binding to these specific sites and facilitate antibody evasion for these XBB variants.

## Data Availability

The simulation systems and trajectories will be made available upon request.

## Supplementary Materials

The following supporting information can be downloaded at: https://www.mdpi.com/article/10.3390/ijms241612586/s1.
