# Supplementary material for: Distinctive Features of the XBB.1.5 and XBB.1.16 Spike Protein Receptor-Binding Domains and Their Roles in Conformational Changes and Angiotensin-Converting Enzyme 2 Binding
Source: Int J Mol Sci. 2023 Aug 9;24(16):12586. doi: 10.3390/ijms241612586 (PMC10454900; doi:10.3390/ijms241612586)
Supplement: Supplementary file 1 [file ijms-24-12586-s001.zip › Supplementary_Figures S1-S4 and Table S1.pdf]

Supplementary Material

Distinctive features of the XBB.1.5 and XBB.1.16 Spike Protein RBDs and their roles in conformational changes and ACE2 binding

Tej Sharma [1], Bernard Gerstman[1,2] and Prem Chapagain[1,2,*]

[1] Department of Physics, Florida International University, Miami, FL 33199, USA
[2] Biomolecular Sciences Institute, Florida International University, Miami, FL 33199, USA
* Correspondence: chapagap@fiu.edu; Tel.: +1 305 348 6266

Table S1
Figure S1
Figure S2
Figure S3
Figure S4
Video S1
Video S2

Table S1. List of all molecular systems used in this work. The WT, delta and omicron simulations of the RBD-only, RBD-hACE2, and WT and Omicron RBD-down Trimer systems are from earlier work (indicated by one *) and all others are new simulations.

System	Variant/ (PDB ID)	# Water	Ion Conc.	# Atoms (total)	Box Size (Å)^3	Time (ns)
RBD Only	XBB.1.5 (Mutated on 7T9L)	28,360	0.15 M	835,23	96x96x96	1000
	XBB.1.16 (Mutated on 7T9L)	24,76 5	0.15 M	72,749	92x92x92	1000
RBD-hACE2	Delta* (7W9I)	75,956	0.15 M	242,060	138x138x138	200
	Omicron* (7T9L)	70,801	0.15 M	226,700	134x134x134	200
	XBB.1.5 (Mutated on 7T9L)	82,424	0.15 M	242,286	137x137x137	300
	XBB.1.5 Run 2**	82,424	0.15 M	242,286	137x137x137	100
	XBB.1.16 (Mutated on 7T9L)	77,837	0.15 M	247,015	144x144x144	300
	XBB.1.16 Run 2**	77,140	0.15 M	246,725	144x144x144	100
	XBB.1.16 Run 3**	77,155	0.15 M	246,205	141x141x141	100
RBD-down	XBB.1.5/XBB.1.16*** RBD-down Trimer	89,355	0.15 M	261,559	141x141x141	100

*

Simulations extended from 100 to 200ns from ref. Hossen, M.L., et al. *PCCP* **24**, 9123-9129 (2022).

** Rerun from the last frame of 200 ns of XBB.1.5 (mutated on 7T9L).

***Residues in the truncated systems: Chain A (330-530); Chain B (30-530, 968-1000); Chain C (330-530, 968-1000).

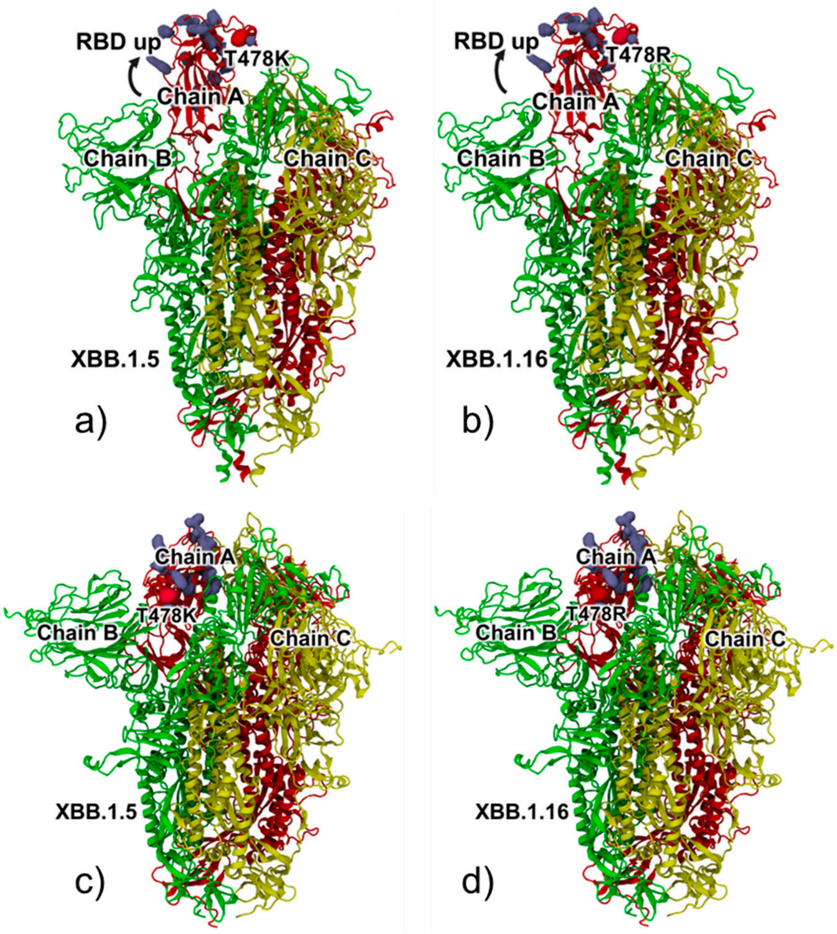

Figure S1. The RBD-up conformation for the spike trimer, showing the mutations in the RBD of chain A of a) XBB.1.5 and b) XBB.1.16. The RBD-down conformation for c) XBB.1.5 and d) XBB.1.16, showing the same mutations as in a-b. The T478K/R mutations highlighted in red surface.

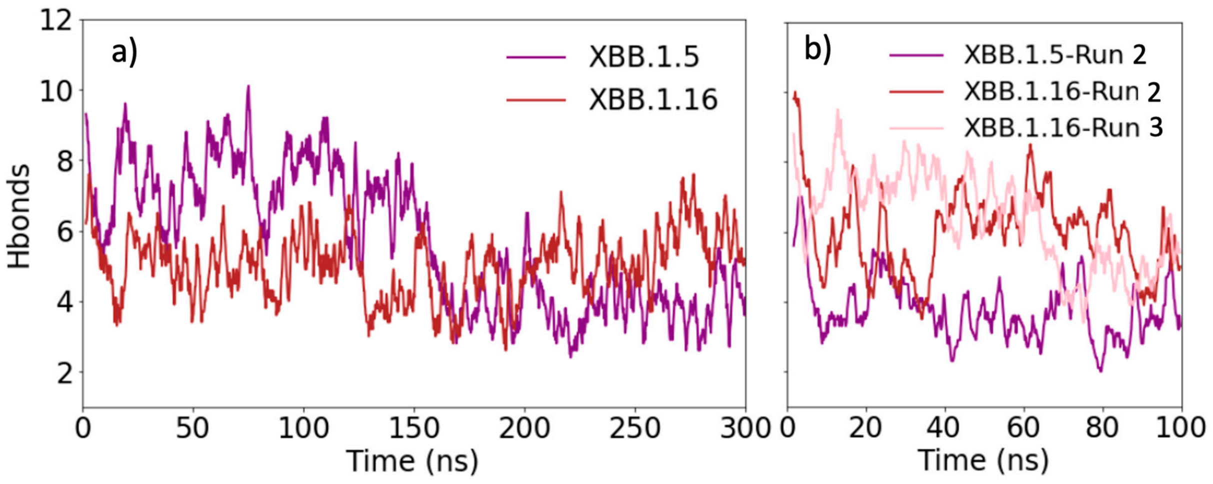

Figure S2. Number of hydrogen bonds between RBD and ACE2 for XBB.1.5 and XBB.1.16 as a function of time. a) For both XBB.1.5 and XBB.1.16, mutations were introduced to omicron RBD. Therefore, the simulations started from the structure of the omicron RBD-ACE2 complex. b) Reruns of XBB.1.5 and XBB.1.16, starting from the frame extracted at 200-ns of the XBB.1.5 of a). Even though both reruns of XBB.1.16 were started from the XBB.1.5 structure, hydrogen bonds were restored, whereas the number of hydrogen bonds remained lower in the rerun of XBB.1.5.

a) XBB.1.5 Run 2

	Q24	K31	H34	E35	D38	Y83	K353	D355
Y449					10			
Y453			14					
A475	13							
N487						32		
Q493		10		51				
R498					11			
T500								104
G502							82	

b) XBB.1.16 Run 2

	S19	Q24	K31	H34	E35	D38	Q42	Y83	K353	D355
Y449						27				
Y453				81						
A475	24	19								
N477	31									
N487								51		
S490			13							
Q493			30	29	50					
R498							10			
T500										100
G502									85	

c) XBB.1.16 Run 3

	S19	Q24	T27	D30	K31	H34	E35	Y83	K353	D355
N417				26						
Y453						72				
A475	30	16						31		
N477	33									
N487								44		
Y489			31							
Q493					15	23	60			
S494						21				
T500										105
Y501									20	
G502									81	

Figure S3. Hydrogen bond occupancies for the interacting residue pairs of RBD and ACE2 for the reruns of Fig. S2. a) XBB.1.5 run 2 a) XBB.1.16 run 2 c) XBB.1.16 run 3.

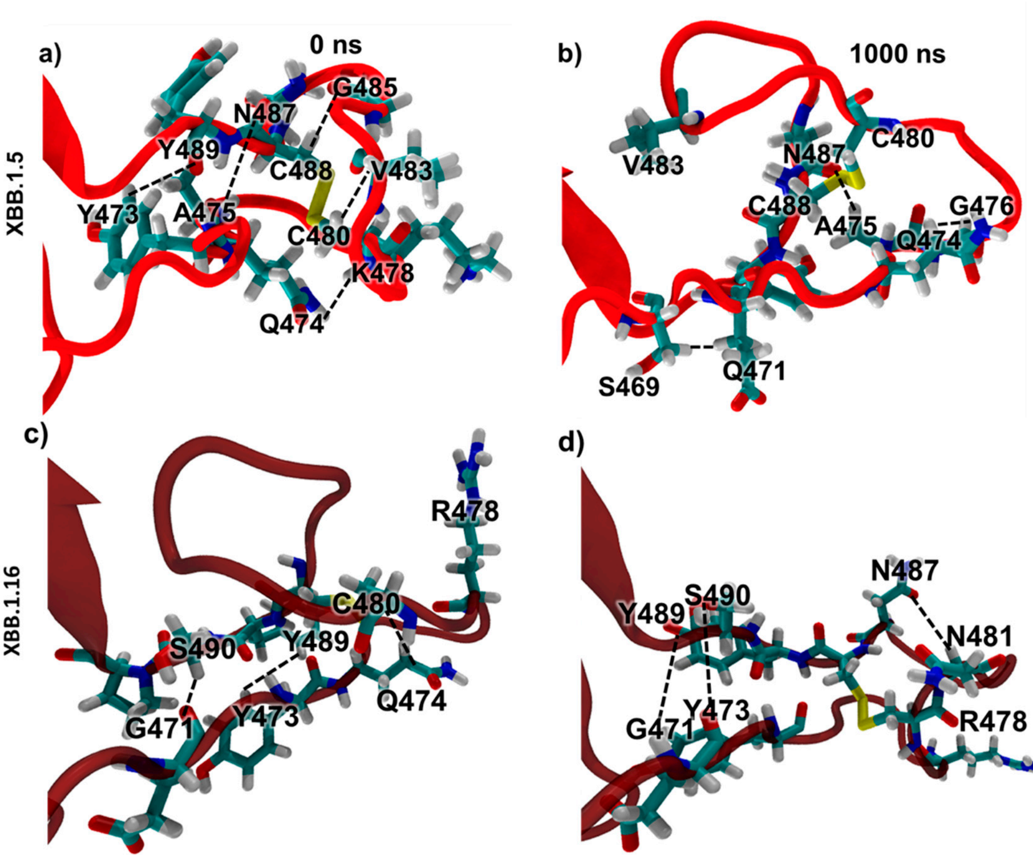

Figure S4. Representative conformations of the a,b) XBB.1.5 and c,d) XBB.1.16 variants displaying hydrogen bonding within the loop residues. The analysis was carried out for the first 20 ns and last 20 ns of a 1000 ns simulation. The initial configuration used for XBB.1.5 was the same as the omicron structure, whereas the initial configuration used for XBB.1.16 was the final configuration from the XBB.1.5 simulation.

Video S1. Conformational dynamics of the Spike protein RBD of XBB.1.5 from a 1000 ns simulation trajectory of the RBD-only system.

Video S2. Conformational dynamics of the Spike protein RBD of XBB.1.16 from a 1000 ns simulation trajectory of the RBD-only system.
